# Mitochondria-targeted peptide elamipretide alleviates cisplatin-induced ovarian failure by activating the nuclear factor erythroid 2-related factor 2/heme oxygenase-1 pathway

**DOI:** 10.3389/fphar.2026.1960111

**Published:** 2026-10-05

**Authors:** Yuhong Zhao, Heqiu Yan, Li Wang, Yu Lei, Ningjing Li, Jun Liu, Xia Bai, Weiwei Zhi, Min Jiang, Li Li, Guohui Zhang, Weixin Liu

**Affiliations:** 1 Center of Women’s and Children’s Health Medical Research and Technology Innovation, Sichuan Provincial Women’s and Children’s Hospital/The Affiliated Women’s and Children’s Hospital of Chengdu Medical College, Chengdu Medical College, Chengdu, China; 2 Key Laboratory of Fertility Preservation and Promotion, Sichuan Provincial Women’s and Children’s Hospital/The Affiliated Women’s and Children’s Hospital of Chengdu Medical College, Chengdu, China; 3 Key Laboratory for Minimally Invasive/Non-Invasive Diagnosis and Treatment of Female Reproductive System Diseases and Lifelong Reproductive Health Protection, Sichuan Provincial Women’s and Children’s Hospital/The Affiliated Women’s and Children’s Hospital of Chengdu Medical College, Chengdu, China; 4 College of Laboratory Medicine, Chengdu Medical College, Chengdu, China; 5 Reproductive Laboratory, Meishan Women and Children’s Hospital, Alliance Hospital of West China Second University Hospital, Meishan, China; 6 School of Medicine and Life Sciences, Chengdu University of Traditional Chinese Medicine, Chengdu, China; 7 Key Laboratory of Reproductive Medicine, Sichuan Provincial Women’s and Children’s Hospital/The Affiliated Women’s and Children’s Hospital of Chengdu Medical College, Chengdu, China

**Keywords:** mitochondrial dysfunction, Nrf2/HO-1 pathway, oxidative stress, premature ovarian failure, SS31

## Abstract

**Background:**

Premature ovarian failure (POF) is a complex endocrine disorder that severely impairs female fertility. Cisplatin, a commonly used chemotherapeutic agent, has been identified as a major risk factor for POF in young women. This study aimed to investigate the protective effects and underlying mechanisms of the mitochondria-targeted peptide elamipretide (SS31) in a cisplatin-induced mouse model of POF.

**Methods:**

POF was established via intraperitoneal cisplatin (2 mg/kg, 7 days), followed by SS31 treatment (10 mg/kg, 3 weeks).

**Results:**

SS31 treatment significantly alleviated cisplatin-induced ovarian damage and reversed key pathological features of POF. Mice treated with SS31 exhibited more rapid recovery in body weight, increased ovarian size and weight, improved ovarian morphology, and a significantly higher number of follicles at various developmental stages compared to the model group. SS31 also normalized serum hormone levels, reduced oxidative stress, restored mitochondrial membrane potential, and improved mitochondrial morphology in oocytes and granulosa cells. Mechanistically, SS31 activated the nuclear factor erythroid 2-related factor 2 (Nrf2)/heme oxygenase-1 (HO-1) signaling pathway in ovarian tissues.

**Conclusion:**

The mitochondria-targeted peptide SS31 effectively ameliorates cisplatin-induced POF in mice by enhancing antioxidant defenses and preserving mitochondrial function. These findings suggest that SS31 is a promising therapeutic candidate for the clinical management of POF and provide new insights into antioxidant-based interventions.

## 1 Introduction

Premature ovarian failure (POF) is a gynecological endocrine disorder characterized by highly heterogeneous etiologies. It refers to the onset of amenorrhea in women under the age of 40, accompanied by elevated serum levels of follicle-stimulating hormone (FSH) and decreased levels of estradiol (E_2_), as well as varying degrees of perimenopausal symptoms. POF significantly impacts women’s health and quality of life (Welt, 2008). The incidence of POF is rising annually and currently affects approximately 3.5% of women of reproductive age worldwide. It is one of the leading causes of infertility in this population, yet effective therapies to restore ovarian function remain limited (Li M. et al., 2023). Patients with POF typically exhibit reduced oocyte quantity and quality, leading to lower clinical pregnancy and live birth rates, thereby negatively influencing reproductive outcomes (Hu et al., 2020). The pathogenesis of POF is believed to involve accelerated follicular atresia or dysregulation in the activation and recruitment of primordial follicles, resulting in premature follicle depletion and impaired folliculogenesis (De Vos et al., 2010). Mitochondrial dysfunction and reactive oxygen species (ROS)-induced oxidative damage have also been implicated in the decline of oocyte quality and quantity in POF patients (Lin et al., 2022).

The etiology of POF is multifactorial and includes genetic, autoimmune, iatrogenic (e.g., chemotherapy), and environmental factors (Laml et al., 2000). Among them, cancer therapies such as chemotherapy and radiotherapy are recognized as major contributors to ovarian dysfunction and infertility in reproductive-age women, owing to the high sensitivity of ovarian reserve to these treatments. Hence, developing strategies to preserve ovarian function during chemotherapy is of great clinical importance. Cisplatin (CIS) is a widely used chemotherapeutic agent with known gonadotoxicity, limiting its application in young female patients. CIS exerts its cytotoxic effects by disrupting DNA structure and inducing apoptosis in normal ovarian follicles, which compromises oocyte structure and function. Moreover, it elevates ROS production, triggering oxidative stress in ovarian tissues (Zhang et al., 2023). Compared with the general population, women under 40 who undergo cancer treatment face a 38% reduction in the probability of conception (Anderson et al., 2018). Therefore, interventions aimed at preserving fertility in young cancer patients are urgently needed. Notably, animal models of POF induced by CIS closely replicate the histological and endocrine features observed in human patients, while also offering advantages such as low mortality, short intervention periods, and cost-effectiveness (Da et al., 2023).

In recent years, increasing evidence has shown that antioxidants play a pivotal role in protecting ovarian function. Oral antioxidants such as coenzyme Q10 and chitosan oligosaccharide-zinc have been reported to reduce ROS-mediated oocyte damage (Delkhosh et al., 2019; Li et al., 2021). However, their therapeutic efficacy is often limited due to poor membrane permeability, lack of targeting ability, short half-life, and low bioavailability—issues compounded by gastrointestinal factors such as solubility and dissolution rate (Banerjee and Pillai, 2019). Therefore, the development of novel mitochondria-targeted antioxidants holds significant promise for clinical interventions in POF.

Elamipretide (SS31) is a novel mitochondria-targeted tetrapeptide with a unique molecular structure that allows it to selectively accumulate in the inner mitochondrial membrane. SS31 functions as a potent mitochondrial protectant and antioxidant. It freely crosses cellular and mitochondrial membranes without the need for transport proteins or carriers, and specifically binds to cardiolipin, thereby inhibiting cytochrome c oxidation, stabilizing mitochondrial membrane potential, and preserving the integrity of the electron transport chain. The dimethyltyrosine moiety of SS31 reacts with oxygen free radicals to form inactive tyrosyl radicals and subsequently dityrosine, enabling efficient ROS scavenging and cellular protection (Wu et al., 2015; Szeto, 2014). Compared to conventional antioxidants such as coenzyme Q10, astaxanthin, and vitamin E, SS31 achieves higher local concentrations at the primary site of ROS production—within mitochondria. Consequently, SS31 exhibits strong antioxidative, anti-apoptotic, and cytoprotective effects, and has shown therapeutic efficacy in models of heart, brain, and kidney diseases (Szeto, 2014; Du et al., 2024), with ongoing clinical trials. Although SS31 has been investigated in the treatment of mitochondrial cardiolipin metabolism disorders and ischemic diseases, its application in reproductive system disorders such as POF has not yet been reported. Prior studies have confirmed its high bioavailability, metabolic stability, and sustained *in vivo* activity, suggesting its potential as a therapeutic agent for POF (Szeto, 2014; Zhu et al., 2022).

This study aimed to investigate the therapeutic potential of SS31 in a mouse model of CIS-induced POF. We evaluated the efficacy of SS31 in restoring ovarian function and explored its underlying mechanisms. Our findings may offer a novel, feasible strategy for fertility preservation in women at risk of chemotherapy-induced ovarian failure and provide a theoretical basis for future clinical applications.

## 2 Materials and methods

### 2.1 Establishment of a POF mouse model and SS31 treatment

Eight-week-old female SPF Kunming (KM) mice were purchased from Beijing Huafukang Bioscience Co., Ltd.: Beijing, China. Animals were housed in the Animal Experiment Center of Sichuan Maternal and Child Health Hospital under a controlled temperature (24 °C ± 1 °C) and a 12-h light/dark cycle in a pathogen-free environment, with free access to food and water.

Female mice were randomly assigned to three groups: control, CIS, and SS31-treated. To establish the POF model, mice in the model and SS31-treated groups received intraperitoneal (i.p.) injections of cisplatin (2 mg/kg/day; MCE, HY-17394) daily for seven consecutive days (days 1–7), whereas control mice received an equal volume of 0.9% saline during the same period. From days 8–28, mice in the control and model groups continued to receive daily i.p. injections of saline, while mice in the SS31-treated group received daily i.p. injections of SS31 (10 mg/kg/day) (Figure 1A). Body weights were recorded on days 0, 7, and 28.

**FIGURE 1:**
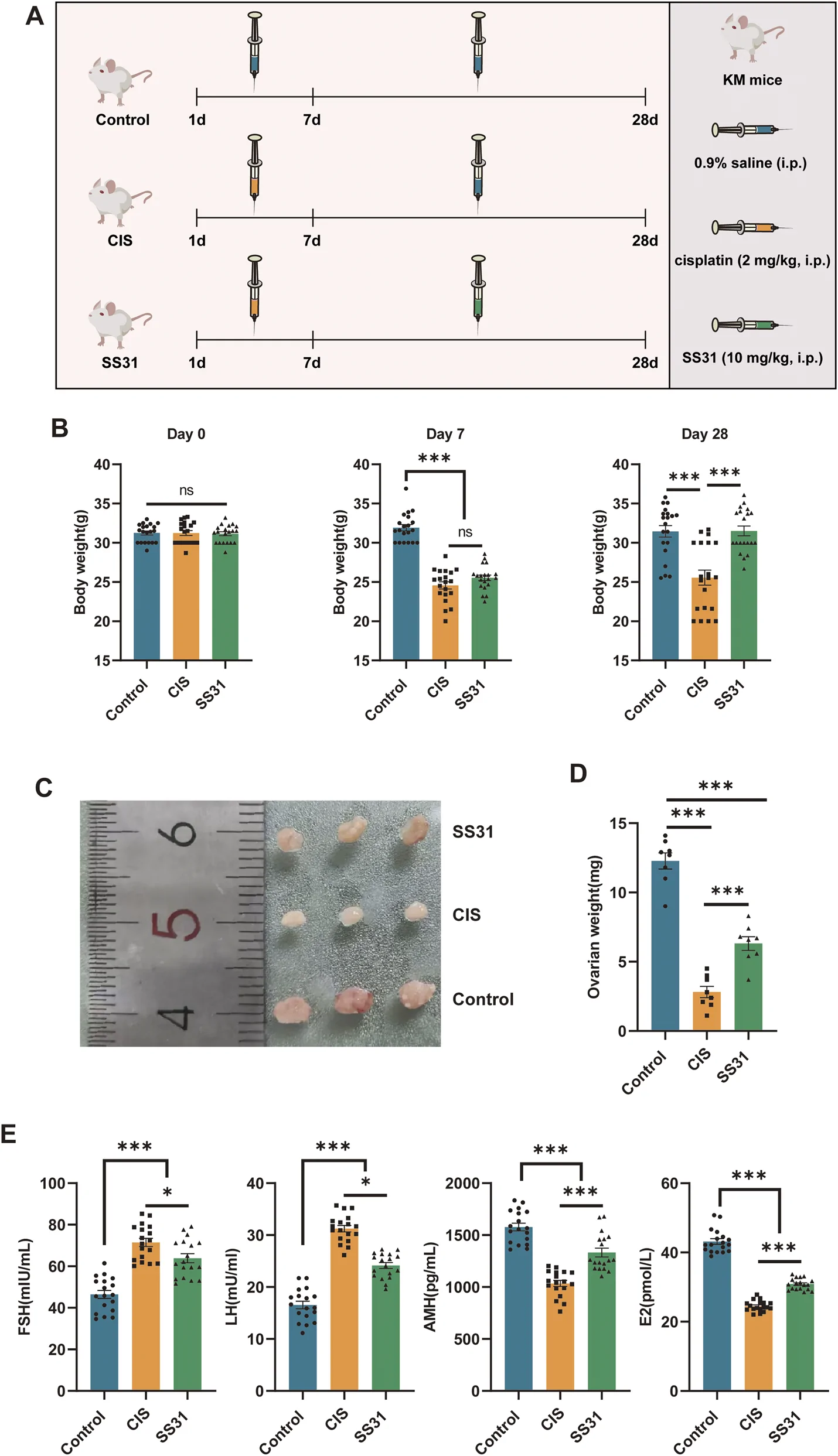
SS31 treatment alleviates cisplatin-induced ovarian damage in POF mice. **(A)** Schematic diagram of the animal experiment: mice were intraperitoneally injected with CIS (2 mg/kg) for seven consecutive days, followed by SS31 treatment (10 mg/kg) for 21 days, after which analyses were performed. **(B)** Quantitative analysis of body weight in control, cisplatin, and SS31-treated POF mice at days 0, 7, and 28 (N = 20). **(C)** Comparison of isolated ovarian volumes among the three groups (N = 3). **(D)** Quantification of ovarian weights from the three groups (N = 8). **(E)** Serum levels of FSH, LH, AMH, and E_2_ in the three groups (N = 18). (*P < 0.05, ***P < 0.001; ns: not significant).

### 2.2 Blood collection and hormone measurement

At day 7 (post-cisplatin) and day 28 (after SS31 treatment), blood samples were collected via retro-orbital puncture into 2 mL centrifuge tubes (Parasuraman et al., 2010). After clotting for 30 min at room temperature, the samples were centrifuged at 3,000 rpm for 15 min. The serum supernatant was collected, centrifuged again at 3,000 rpm for 15 min, and stored at −80 °C for further analysis. Serum levels of FSH, luteinizing hormone (LH), anti-Müllerian hormone (AMH), and E_2_ were measured using ELISA kits according to the manufacturer’s instructions (Ruixin Bio: FSH, RX203000M; LH, RX202015M; AMH, RX202731M; E_2_, RXJ203008M).

### 2.3 Histological staining and follicle counting

After blood collection, mice were sacrificed via cervical dislocation. Bilateral ovaries were harvested and fixed in 4% paraformaldehyde for at least 24 h, embedded in paraffin, and sectioned at 5 μm thickness (Li and Kuang, 2024). Hematoxylin and eosin (H&E) staining was performed to evaluate ovarian morphology and follicle development. Only follicles containing a visible oocyte nucleus were counted. Follicles were classified as follows: Primordial follicles: oocytes surrounded by a single layer of flattened granulosa cells; Primary follicles: surrounded by a single layer of cuboidal granulosa cells; Secondary follicles: more than two layers of cuboidal granulosa cells; Antral follicles: presence of antral cavities between granulosa cells and the oocyte (Myers et al., 2004).

### 2.4 Oxidative stress assessment in ovarian tissues

Ovarian tissues were homogenized in appropriate lysis buffers, then centrifuged at 12,000 rpm for 5–10 min. The supernatant was used to determine levels of oxidative stress markers including malondialdehyde (MDA; Beyotime: Shanghai, China, S0131S) (Rui et al., 2021), hydrogen peroxide (H_2_O_2_; Beyotime, S0038) (Mu et al., 2021), total antioxidant capacity (T-AOC; Nanjing Jiancheng, A015-2-1) (Luo et al., 2021), superoxide dismutase (SOD; Nanjing Jiancheng, A001-3) (Loh et al., 2010), and glutathione peroxidase (GPx; Beyotime, S0057S) (Tao et al., 2023). Measurements were performed with a microplate reader (BioTek Epoch), and protein concentrations were determined using a BCA assay kit (Lablead: Beijing, China, B5001) (Qin et al., 2024). Standard curves were generated to calculate absolute concentrations.

### 2.5 Terminal deoxynucleotidyl transferase-mediated dUTP nick-end labeling (TUNEL) staining of ovarian sections

Paraffin-embedded ovarian sections were deparaffinized and rehydrated, then subjected to TUNEL staining using a commercial kit (Vazyme, A111-03) to assess apoptosis. Nuclei were counterstained with DAPI (Servicebio, G1012-100ML). Images were captured under a fluorescence microscope, and the number of TUNEL-positive cells per follicle was quantified (Gavrieli et al., 1992; Xie et al., 2025).

### 2.6 Transmission electron microscopy (TEM)

Ovarian tissues were fixed in electron microscopy fixative (Servicebio, G1102-100ML), dehydrated through graded acetone series, infiltrated with embedding reagents (Epon-812), and embedded. Semithin sections were examined under a light microscope to identify secondary oocyte areas. Ultrathin sections (60–90 nm) were cut and placed on copper grids, stained with uranyl acetate (10–15 min) and lead citrate (1–2 min), and observed using a transmission electron microscope (JEOL JEM-1400FLASH). Mitochondrial ultrastructure in oocytes and granulosa cells was examined at 25,000× magnification. For each group, six electron microscopy sections were analyzed. Within each sample, 100 mitochondria were randomly counted from oocytes and another 100 from granulosa cells. The proportion of abnormal mitochondria (%) was calculated (Chen et al., 2025; Reynolds, 1963).

### 2.7 Superovulation and oocyte mitochondrial membrane potential detection

Mice were superovulated by i.p. injection of 10 IU pregnant mare serum gonadotropin, followed by 10 IU human chorionic gonadotropin after 48 h. After 14–16 h, mice were sacrificed, and cumulus-oocyte complexes (COCs) were collected from the ampulla of the oviduct (Mara et al., 2020). Cumulus cells were removed using 0.5 mg/mL hyaluronidase (Solarbio: Beijing, China, H8030) (Contreras et al., 2022). Mitochondrial membrane potential was assessed using the 5,5′,6,6′-tetrachloro-1,1′,3,3′-tetraethylbenzimidazolylcarbocyanine iodide (JC-1) probe (1:200; Beyotime, C2003S) (Lyu et al., 2022), and nuclei were counterstained with Hoechst 33,342 (1:100; Beyotime, C1029) (Gu et al., 2019). Confocal microscopy was used for image acquisition. Red/green fluorescence intensity ratios were calculated using ImageJ.

### 2.8 Western blot analysis

Ovarian tissues were lysed in pre-cooled RIPA buffer (Beyotime, P0013B) on ice for 30 min, followed by centrifugation (12,000 rpm, 4 °C, 5 min). Protein concentration was determined using a BCA assay (Lablead, B5001). Samples were mixed with 5× SDS-PAGE loading buffer, denatured at 95 °C for 10 min, and separated using 4%–12% precast SDS-PAGE gels (Lablead, P41212). Proteins were transferred onto polyvinylidene difluoride membranes (Millipore: Billerica, MA, United States, ISEQ00010), blocked for 1 h, and incubated overnight with primary antibodies at 4 °C, followed by HRP-conjugated secondary antibodies for 1 h at room temperature. Bands were visualized using enhanced chemiluminescence (Lablead, E1060) and analyzed using ImageJ.

Primary antibodies used: Caspase-3 (Abclonal: Wuhan, China, A21677, 1:1,000), Bax (Abclonal, A20227, 1:500), Bcl-2 (Abclonal, A20777, 1:1,000), NRF2 (Abclonal, A21176, 1:1,000), HO-1 (Proteintech: Rosemont, IL, United States, 10701-1-AP, 1:2,000), α-Tubulin (Abclonal, A6830, 1:2,000), GAPDH (Abclonal, A19056, 1:10,000).

Secondary antibodies used: HRP-conjugated Donkey anti-Mouse IgG (Abclonal, AS033, 1:5,000), HRP-conjugated Goat anti-Rabbit IgG (Abclonal, AS014, 1:5,000).

### 2.9 Reverse transcription-quantitative polymerase chain reaction (RT-qPCR)

Total RNA was extracted from ovarian tissues using the RNA Easy Fast Tissue/Cell Kit (TIANGEN: Beijing, China, DP451) and reverse-transcribed into cDNA using the ABScript Neo RT Master Mix with gDNA Remover (ABclonal, RK20433). RT-qPCR was performed using 2× Universal SYBR Green Fast qPCR Mix (ABclonal, RK21203) on a real-time PCR system. Primer sequences were synthesized by Beijing Qingke Biotechnology Co., Ltd. (listed in Supplementary Table S1). Gene expression was normalized to β-actin, and relative expression was calculated using the 2^−ΔΔCT^ method (Xu et al., 2026; Wu et al., 2025).

### 2.10 Statistical analysis

Data distribution was assessed using the Shapiro-Wilk test, and homogeneity of variances was evaluated by Bartlett’s test. For datasets meeting both assumptions, one-way ANOVA followed by Tukey’s *post hoc* test was applied. For datasets with unequal variances (Bartlett’s test, P < 0.05), Welch’s ANOVA with Dunnett’s T3 *post hoc* test was used instead. All data are presented as mean ± SEM, and P < 0.05 was considered statistically significant. GraphPad Prism 8.0 was used for statistical analysis and graph generation.

## 3 Results

### 3.1 SS31 intervention attenuates cisplatin-induced ovarian injury in POF mice

Body weights of mice in each group were recorded on days 0, 7, and 28 to assess overall health status (Figure 1B). No significant differences in initial body weights were observed among groups on day 0. However, after 7 days of CIS administration, mice in the CIS-treated groups exhibited significant weight loss and deteriorated condition compared to controls. By day 28, SS31 treatment markedly improved body weight, alleviating CIS-induced weight reduction. Gross morphological examination of excised ovaries revealed a significant reduction in ovarian volume following CIS treatment, whereas SS31 administration effectively restored ovarian size (Figure 1C). Ovarian weight displayed a consistent trend, with SS31 significantly reversing CIS-induced ovarian weight loss (Figure 1D). Since ovarian size and weight alone cannot fully reflect ovarian function, serum hormone levels were measured to evaluate gonadal status. Compared to controls, CIS treatment resulted in elevated serum FSH and LH levels and decreased AMH and E_2_ levels. SS31 treatment significantly normalized these hormone levels (Figure 1E).

Histological examination is a direct indicator of ovarian damage. H&E staining (Figure 2A) showed numerous follicles at various developmental stages in control ovaries, with intact tissue architecture and orderly cell arrangement. In contrast, CIS-treated ovaries exhibited markedly reduced size, increased follicular atresia, and decreased follicle counts, consistent with ovarian volume and weight data. SS31 treatment significantly alleviated ovarian damage and follicle loss induced by CIS. Quantitative follicle counting revealed that SS31 significantly restored the numbers of primordial, primary, and antral follicles reduced by CIS treatment (Figure 2B). To further assess follicle development, COCs obtained after superovulation were counted. CIS treatment significantly decreased COCs numbers compared to controls, while SS31 intervention partially restored COCs yield (Figure 2C), suggesting enhanced follicular development capacity.

**FIGURE 2:**
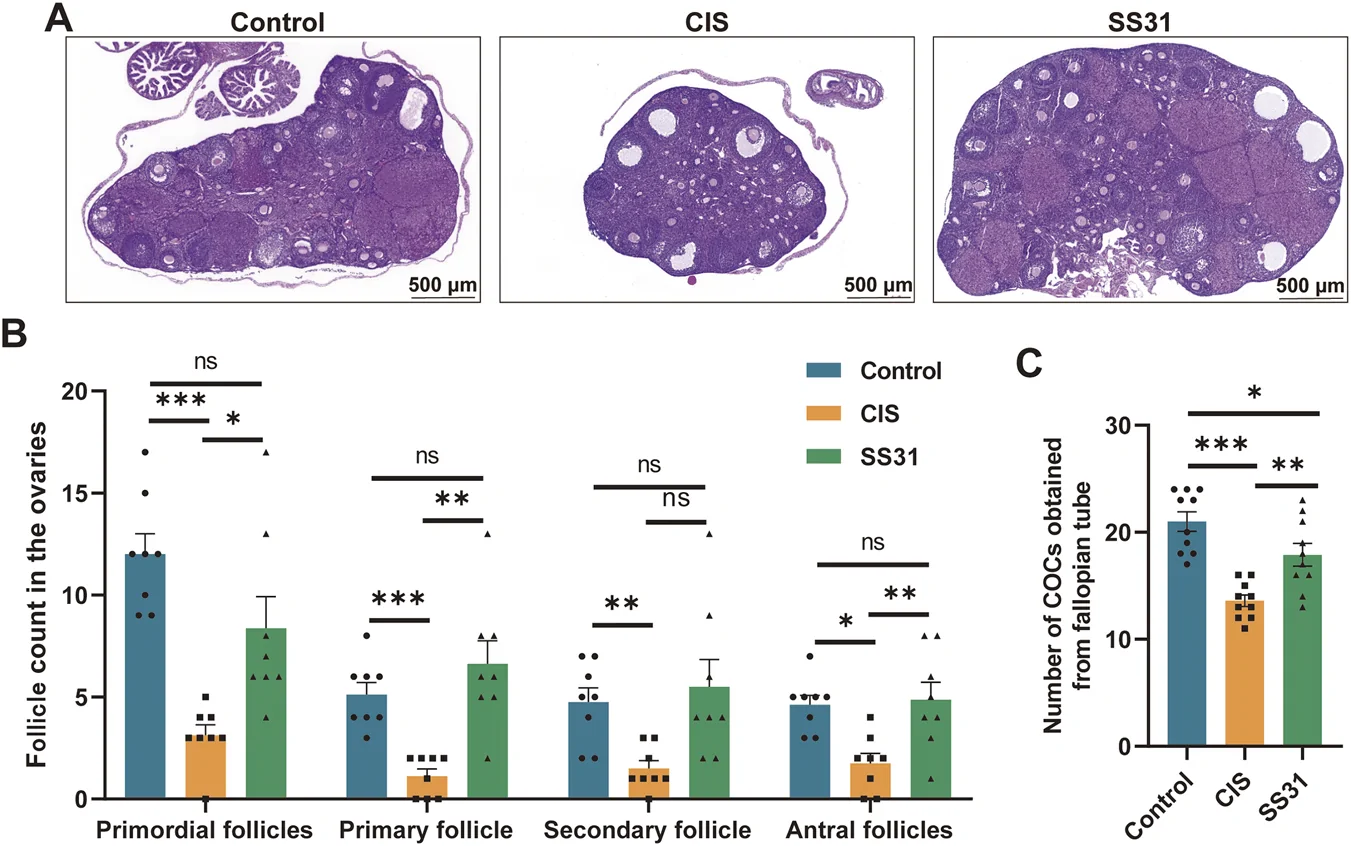
SS31 improves ovarian morphology and follicle counts in cisplatin-induced POF mice. **(A)** Representative H&E staining of ovarian sections from control, CIS, and SS31-treated mice. Scale bar: 500 μm. **(B)** Quantitative analysis of primordial, primary, secondary, and antral follicles in ovaries of the three groups (N = 8). **(C)** Number of COCs collected after superovulation in each group (N = 4). (*P < 0.05, **P < 0.01, ***P < 0.001; ns: not significant).

### 3.2 SS31 enhances antioxidant capacity and reduces oxidative damage in POF ovaries

As a mitochondria-targeted antioxidant, SS31 showed protective effects against CIS-induced oxidative damage in ovaries. ELISA assays measured levels of MDA, H_2_O_2_, T-AOC, SOD, and GPx in ovarian tissue (Figure 3). CIS treatment significantly increased MDA and H_2_O_2_ levels, indicating exacerbated lipid peroxidation and oxidative stress, while SS31 treatment markedly reduced these markers (Figures 3A,B). Furthermore, CIS decreased T-AOC, SOD, and GPx levels, which were partially restored by SS31 intervention (Figures 3C–E), indicating enhanced antioxidant defense.

**FIGURE 3:**
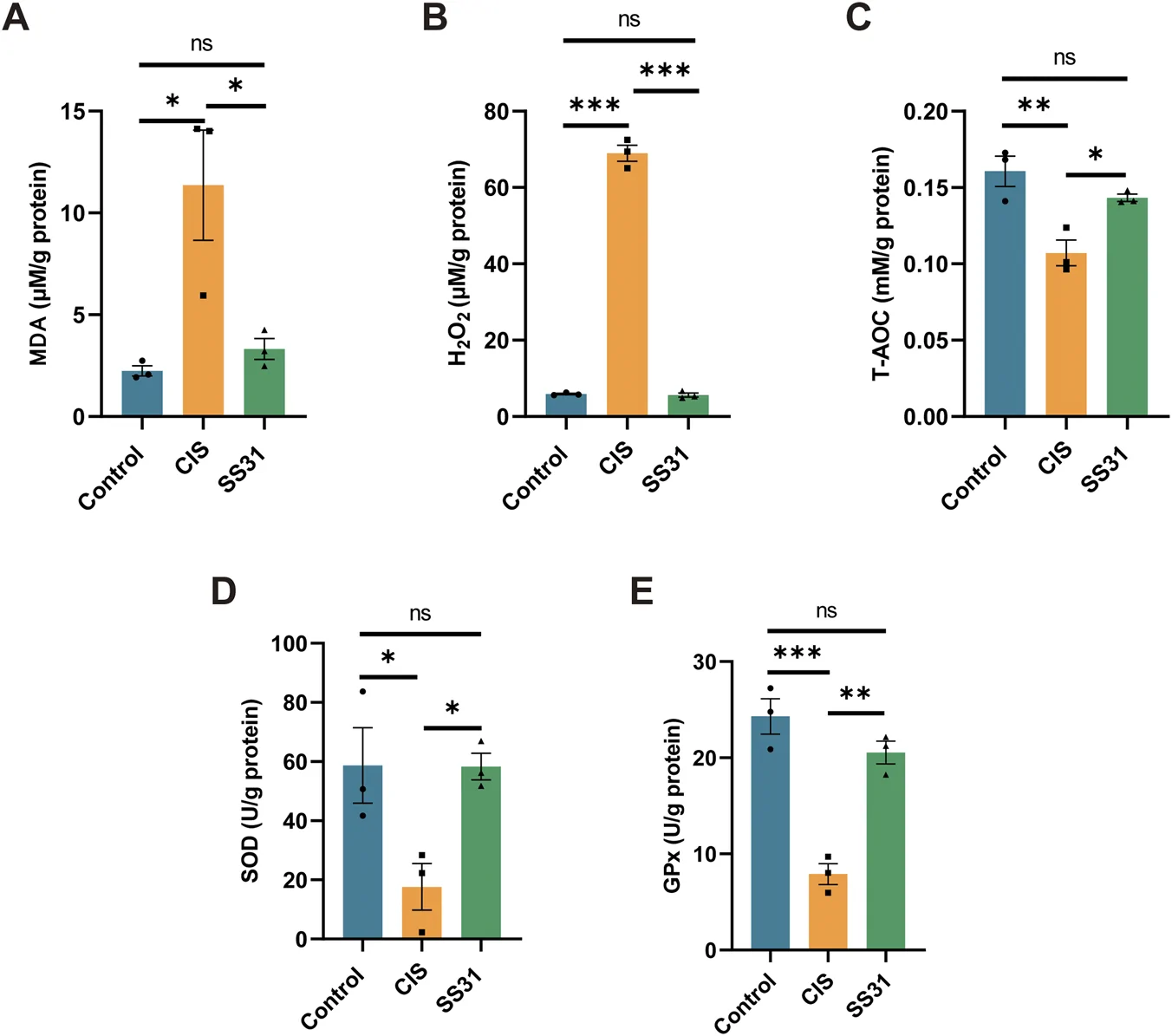
SS31 enhances antioxidant capacity and reduces oxidative damage in ovaries of POF mice. **(A)** Malondialdehyde (MDA) levels in ovarian tissues (N = 3). **(B)** hydrogen peroxide (H_2_O_2_) content in ovarian tissues (N = 3). **(C)** Total antioxidant capacity (T-AOC) in ovarian tissues (N = 3). **(D)** Superoxide dismutase (SOD) activity in ovarian tissues (N = 3). **(E)** Glutathione peroxidase (GPx) activity in ovarian tissues (N = 3). (*P < 0.05, **P < 0.01, ***P < 0.001; ns: not significant).

### 3.3 SS31 reduces apoptosis in ovarian cells of POF mice

TUNEL staining was used to assess ovarian cell apoptosis. As shown in Figure 4A, CIS-treated ovaries exhibited significantly more TUNEL-positive apoptotic cells, whereas SS31 treatment notably decreased apoptotic cell numbers. Quantitative analysis confirmed that SS31 significantly inhibited CIS-induced apoptosis (Figure 4B).

**FIGURE 4:**
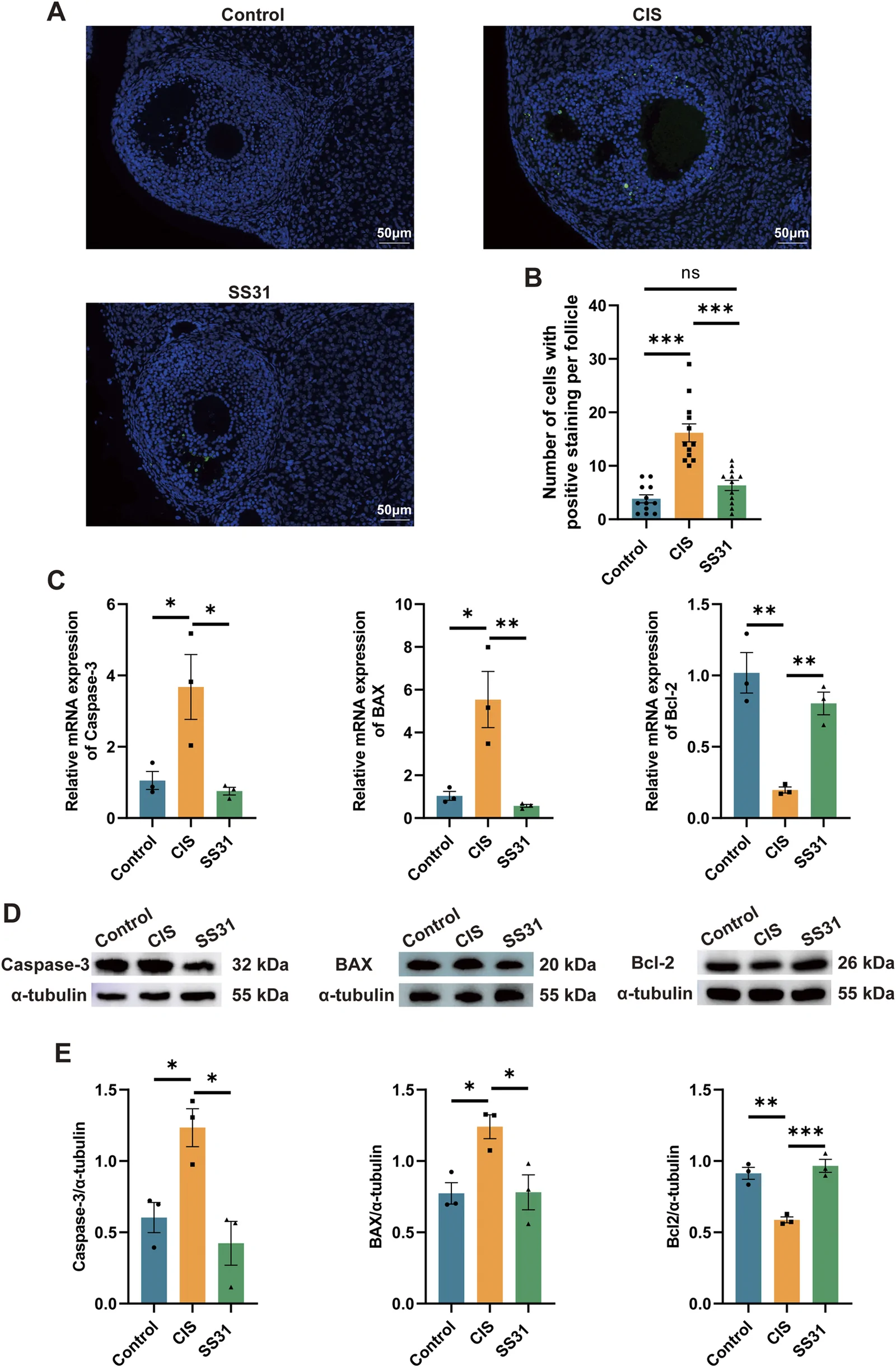
SS31 reduces ovarian cell apoptosis in cisplatin-induced POF mice. **(A)** Representative terminal deoxynucleotidyl transferase-mediated dUTP nick-end labeling (TUNEL) staining of ovarian sections from the three groups. Scale bar: 50 μm. **(B)** Quantification of TUNEL-positive apoptotic cells in ovarian follicles (N = 12). **(C)** mRNA expression levels of apoptosis-related genes Caspase-3, Bax, and anti-apoptotic gene Bcl-2 in ovarian tissues (N = 3). **(D)** Western blot analysis of Caspase-3, Bax, and Bcl-2 protein expression. **(E)** Densitometric analysis of the corresponding protein bands (N = 3). (*P < 0.05, **P < 0.01, ***P < 0.001; ns: not significant).

RT-PCR analysis of pro-apoptotic genes cysteine-aspartic protease 3 (Caspase-3) and BCL2-associated X protein (Bax), and anti-apoptotic gene B-cell lymphoma 2 (Bcl-2) revealed that CIS increased Caspase-3 and Bax mRNA levels and decreased Bcl-2 expression. SS31 treatment reversed these changes (Figure 4C). Western blot analysis yielded consistent protein expression patterns (Figures 4D,E), further demonstrating the anti-apoptotic effect of SS31.

### 3.4 SS31 ameliorates mitochondrial damage in ovarian cells induced by CIS

Given that SS31 is a mitochondria-targeted peptide, mitochondrial morphology in oocytes and granulosa cells was examined by transmission electron microscopy. TEM images (Figure 5A) showed intact mitochondria with clear cristae in control mice, whereas CIS-treated mice exhibited swollen mitochondria with decreased electron density, disrupted cristae, and vacuolization. SS31 treatment markedly improved mitochondrial ultrastructure. Quantitative analysis of morphologically abnormal mitochondria revealed a significant increase in the CIS group, which was significantly reduced by SS31 (Figures 5B,C).

**FIGURE 5:**
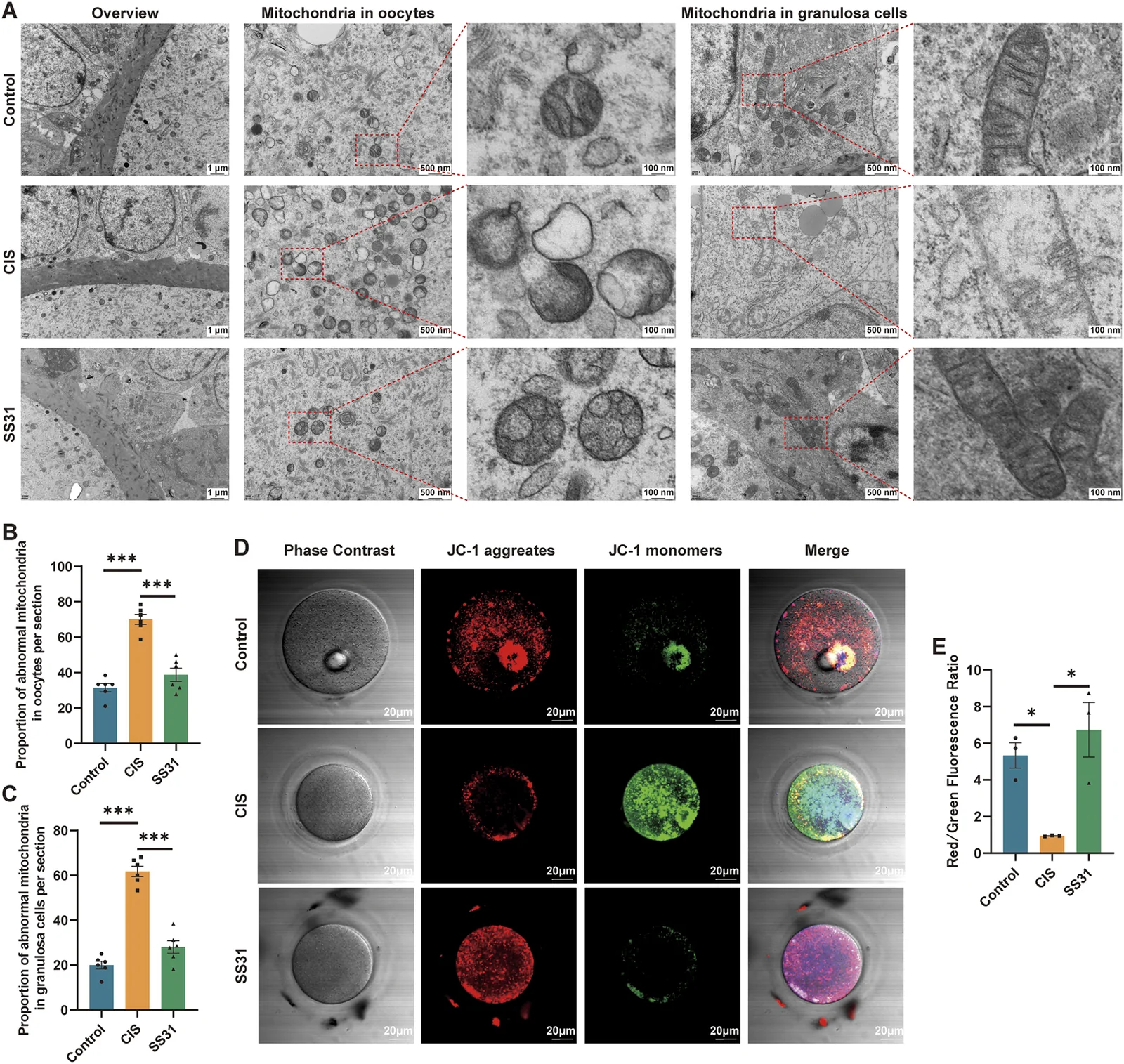
SS31 ameliorates mitochondrial ultrastructural damage in ovaries of cisplatin-induced POF mice. **(A)** Transmission electron microscopy images of secondary follicles from control, CIS, and SS31 groups. Overview: ultrastructure of oocyte, zona pellucida, and granulosa cells in secondary follicles. Mitochondria are visible in cytoplasm of both oocytes and granulosa cells. Scale bars: 1 μm. Mitochondria in oocytes: scale bars, 500 nm. Mitochondria in granulosa cells: scale bars, 500 nm. **(B)** Quantification of morphologically abnormal mitochondria in oocytes (N = 6). **(C)** Quantification of morphologically abnormal mitochondria in granulosa cells (N = 6). **(D)** JC-1 staining of mitochondrial membrane potential in oocytes: The Control group exhibits strong red fluorescence and weak green fluorescence indicating normal membrane potential; CIS group shows weakened red and enhanced green fluorescence, indicating decreased membrane potential and early apoptosis; SS31 treatment restores membrane potential. Scale bars: 20 μm. **(E)** Quantitative analysis of red/green fluorescence ratio in JC-1 stained oocytes (N = 3). (*P < 0.05, ***P < 0.001; ns: not significant).

To further assess mitochondrial function, JC-1 staining of oocytes was performed to measure mitochondrial membrane potential. CIS treatment resulted in a significant decrease in membrane potential, indicative of mitochondrial dysfunction and early apoptosis. SS31 treatment significantly restored membrane potential, as evidenced by increased red/green fluorescence intensity ratio (Figures 5D,E), demonstrating improved mitochondrial function.

### 3.5 SS31 enhances ovarian antioxidant capacity via upregulation of the nuclear factor erythroid 2-related factor 2/heme oxygenase-1 (Nrf2/HO-1) pathway

As described above (Figure 3), SS31 reduced oxidative stress markers MDA and H_2_O_2_ and increased antioxidant enzymes T-AOC, SOD, and GPx, suggesting enhanced antioxidant defense. To investigate the molecular mechanisms underlying these antioxidant effects, we next examined the expression of Nrf2 and its downstream target HO-1. Our results demonstrated that CIS treatment significantly downregulated mRNA and protein expression of Nrf2 and HO-1 in ovarian tissue. SS31 treatment significantly upregulated Nrf2 and HO-1 expression (Figure 6), indicating that SS31 may suppress oxidative stress and improve ovarian function potentially through activation of the Nrf2/HO-1 signaling pathway.

**FIGURE 6:**
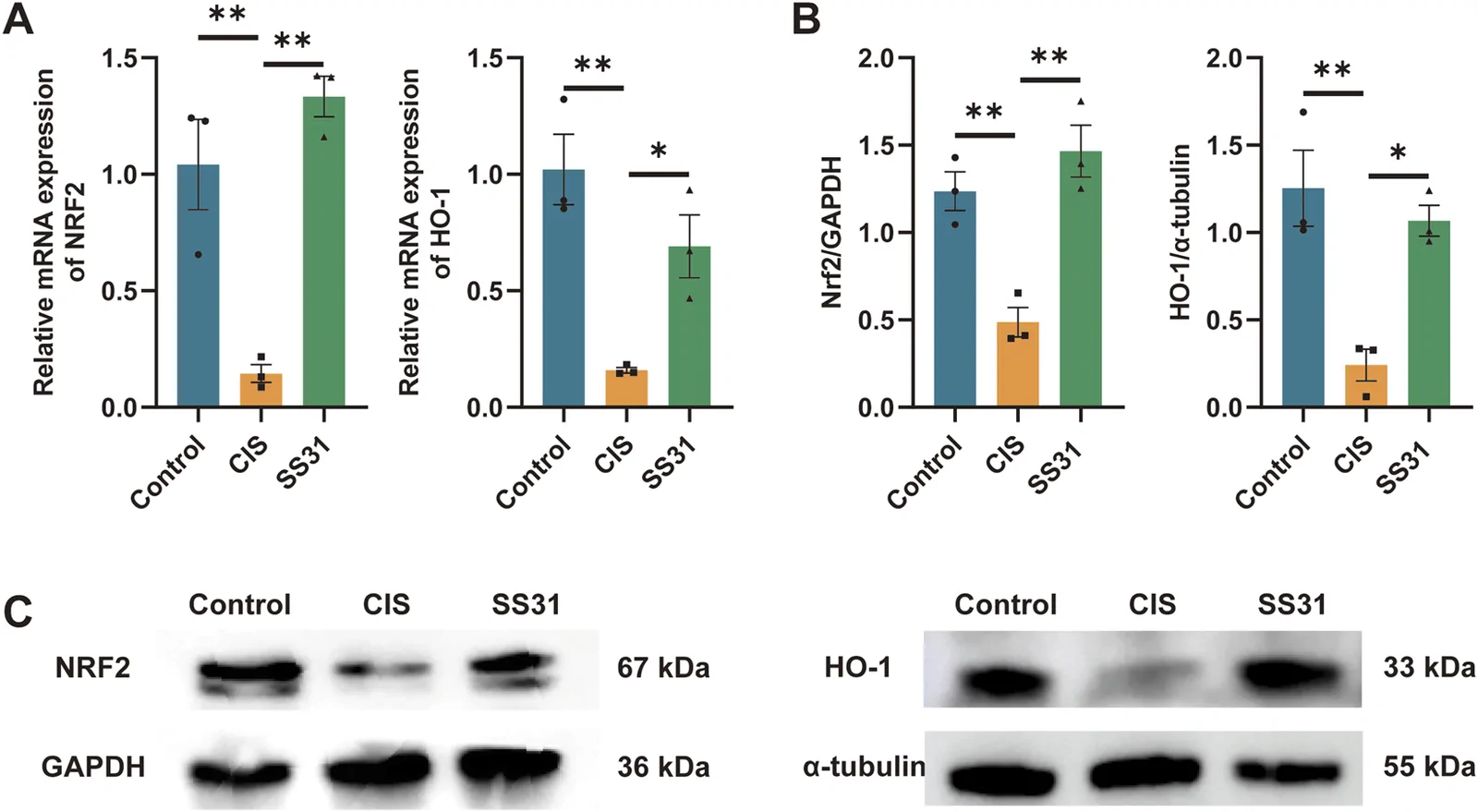
SS31 upregulates the Nrf2/HO-1 signaling pathway to enhance ovarian antioxidant defense. **(A)** mRNA expression levels of Nrf2 and HO-1 in ovarian tissues (N = 3). **(B)** Densitometric quantification of protein expression (N = 3). **(C)** Western blot analysis of Nrf2 and HO-1 protein expression. (*P < 0.05, **P < 0.01).

## 4 Discussion

POF has become one of the leading causes of infertility among women of reproductive age, affecting approximately 3.5% of this population. POF not only significantly impairs female fertility but is also associated with increased risks of cardiovascular disease, osteoporosis, and cognitive decline (Li M. et al., 2023; Ishizuka, 2021). Unlike postmenopausal women, patients with POF often have strong fertility desires; however, effective clinical treatments for this condition remain limited (Cambray et al., 2023; Yuan et al., 2024). Future experimental strategies have been proposed to preserve ovarian follicles from programmed depletion, offering potential avenues for fertility preservation in POF patients (Hegazy, 2020).

With the advancement of chemotherapy, anticancer drugs have been widely used in tumor treatment over recent decades. Since the 1970s, chemotherapy-related adverse effects on female fertility, including amenorrhea and follicular destruction, have been reported (Koyama et al., 1977). The susceptibility of the ovary to damage may stem from similarities between ovarian function characteristics and tumor cell survival mechanisms, as both are major targets of chemotherapy. Since primordial follicles in the ovary lack regenerative capacity and exist in a fixed number, damage to this reserve leads to ovarian dysfunction and premature menopause. Cisplatin, one of the most commonly used chemotherapeutic agents, induces POF by promoting follicular apoptosis and accelerating activation and atresia of primordial follicles (Spears et al., 2019). Cisplatin induces ROS production, depletes intracellular antioxidant reserves, and triggers oxidative stress, ultimately leading to apoptosis. Moreover, cisplatin promotes the release of cytochrome c from mitochondria, activating caspase pathways that result in cellular stress and death (Zhu et al., 2015). Excessive ROS generated by cisplatin predominantly targets mitochondria, causing mitochondrial dysfunction and cell death (Zhang et al., 2023; Nagasawa et al., 2021). Cisplatin also disrupts mitochondrial morphology and function in ovarian cells, leading to decreased mitochondrial membrane potential, reduced ATP synthesis, and increased ROS production (Li Z. et al., 2023). Consequently, antioxidants have been widely recognized as promising agents for preventing or treating chemotherapy-induced tissue damage. Melatonin, as an antioxidant, attenuates ovarian tissue damage, toxic effects, and fertility loss induced by the chemotherapeutic agent cisplatin in mice through its DNA-protective and antioxidant properties (Huang et al., 2021).

In murine studies, the choice of blood collection site is critical, particularly when evaluating hormone-sensitive reproductive endpoints. In the present study, we selected retro-orbital puncture for blood sampling, a technique that is widely used in laboratory rodents due to its ease of performance and ability to yield sufficient blood volumes for subsequent biochemical analyses (Parasuraman et al., 2010). Moreover, compared with the facial vein method, previous studies have demonstrated that repeated facial vein blood collection is associated with more severe clinical adverse events (Frohlich et al., 2018). The tissue processing protocol employed in this study is a conventional method for murine ovarian histological evaluation, which ensures adequate tissue preservation and morphological clarity for accurate follicular counting and structural assessment. Notably, alternative simplified protocols have been developed to reduce processing time and chemical consumption while maintaining comparable histological outcomes (Hegazy and Hegazy, 2015).

SS31, a newly identified mitochondria-targeted antioxidant peptide, specifically binds to cardiolipin on the inner mitochondrial membrane and reduces mitochondrial ROS generation by inhibiting cytochrome c peroxidase activity (Du et al., 2024). Compared to traditional mitochondrial antioxidants such as coenzyme Q10 and SkQ1, SS31’s function is not entirely dependent on mitochondrial membrane potential, resulting in superior therapeutic efficacy. In this study, using an *in vivo* CIS-induced POF mouse model, SS31 demonstrated significant antioxidant effects and improved mitochondrial function. SS31 effectively alleviated oxidative stress and apoptosis in ovarian tissues, restored ovarian morphology, increased follicle quantity and quality, normalized hormonal imbalances, and rescued mitochondrial structure and function. Furthermore, our results suggest that SS31’s protective effects on ovarian tissues may be mediated through upregulation of the Nrf2/HO-1 antioxidant pathway and inhibition of the Bax/Bcl-2/Caspase-3 apoptotic signaling pathway. Previous studies have shown that SS31 reduces MDA levels in mouse ovaries, indirectly reflecting an Nrf2-dependent suppression of lipid peroxidation. The observed decrease in H_2_O_2_ and increase in T-AOC are consistent with typical features of Nrf2 pathway activation. As key antioxidant enzymes, SOD and GPx expression may be regulated by the Nrf2 signaling pathway. The concurrent elevation of SOD, GPx, and T-AOC indicates activation of the Nrf2 pathway (Zhou et al., 2023). These findings collectively suggest that SS31 exerts its antioxidant effects via the Nrf2/HO-1 signaling pathway, enhancing ovarian antioxidant defenses, downregulating caspase-3-mediated apoptotic signals, reducing apoptosis, and thereby improving ovarian morphology and function.

Nrf2 is a ubiquitously expressed antioxidant transcription factor that regulates key metabolic genes and crosstalk with other transcription factors to influence metabolism, antioxidant responses, and mitochondrial function (He et al., 2020). Under oxidative stress, Nrf2 translocates to the nucleus, binds to antioxidant response elements, and activates downstream targets such as HO-1, SOD, and GPx. HO-1, a critical antioxidant enzyme and Nrf2-regulated gene, degrades heme to generate antioxidant molecules like biliverdin and carbon monoxide (Alam et al., 1999). Daphnetin has been reported to significantly ameliorate the premature ovarian failure phenotype by upregulating Nrf2 expression and suppressing NLRP3 inflammasome activation (Zhang et al., 2022). Moreover, the Nrf2/HO-1 axis is recognized as a key signaling pathway that alleviates oxidative stress in cyclophosphamide-induced premature ovarian failure, and its anti-inflammatory and antioxidant properties are essential for mitigating chemotherapy-induced ovarian damage (Sharata et al., 2026). Evidence shows that SS31 reduces oxidative stress by activating Nrf2/HO-1 expression, thereby ameliorating early brain injury following subarachnoid hemorrhage, hypertrophic cardiomyopathy, and oxidative damage and apoptosis in retinal pigment epithelial cells (Zhou et al., 2023; Lu et al., 2017; Bai et al., 2021). Consistent with these reports, our study confirms that SS31 protects against CIS-induced ovarian failure via activation of the Nrf2/HO-1 pathway.

Mitochondria play a decisive role in regulating apoptosis by controlling the release of pro-apoptotic factors. In the mitochondrial apoptotic pathway, cellular stress triggers activation of pro-apoptotic Bcl-2 family proteins such as Bax and downregulation of anti-apoptotic proteins like Bcl-2, leading to cytochrome c release from mitochondria into the cytoplasm, activation of downstream caspase-3, loss of mitochondrial membrane potential, and initiation of apoptosis (Cregan et al., 1999; Shakeri et al., 2017). Caspase-3, a cysteine-aspartic protease, is a key executioner of apoptosis and widely used as a biomarker for apoptotic cell death. Studies in various animal models, including diabetic nephropathy in mice, myocardial ischemia-reperfusion injury in rats, and experimental glaucoma in rats, have shown that SS31 inhibits cytochrome c release, downregulates Bax and caspase-3 expression, upregulates Bcl-2, and restores mitochondrial membrane potential (Hou et al., 2016; Lee et al., 2018; Wu et al., 2019). Our results corroborate these findings, demonstrating that SS31 significantly decreases Bax and caspase-3 expression while increasing Bcl-2 levels.

In summary, this study demonstrates that the mitochondria-targeted peptide SS31 effectively protects against cisplatin-induced premature ovarian failure by reducing oxidative stress and improving mitochondrial function. The underlying mechanisms likely involve upregulation of the Nrf2/HO-1 pathway to enhance antioxidant capacity and inhibition of the caspase-3/Bax/Bcl-2 apoptotic signaling pathway to reduce cell apoptosis, thereby restoring ovarian morphology and function. These findings provide a theoretical basis for the potential use of SS31 as a therapeutic agent for POF.

## 5 Conclusion

In conclusion, our study supports that the mitochondria-targeted peptide SS31 effectively alleviates cisplatin-induced POF in mice by mitigating oxidative stress and preserving mitochondrial integrity of oocytes and granulosa cells. These findings suggest that SS31 may serve as a therapeutic candidate for chemotherapy-induced POF and provide a strong rationale for its clinical translation in fertility preservation. This work highlights the therapeutic potential of targeting mitochondrial dysfunction as a strategy to combat oxidative stress–driven reproductive disorders.

## Data Availability

The raw data supporting the conclusions of this article will be made available by the authors, without undue reservation.
