# Supplementary material for: Mitochondria-targeted peptide elamipretide alleviates cisplatin-induced ovarian failure by activating the nuclear factor erythroid 2-related factor 2/heme oxygenase-1 pathway
Source: Front Pharmacol. 2026 Oct 5;17:1960111. doi: 10.3389/fphar.2026.1960111 (PMC13677905; doi:10.3389/fphar.2026.1960111)
Supplement: Supplementary file 1 [file Table1.docx]

**Table S1 Primers sequences for RT‐qPCR**

| Primers for RT‐PCR (5′–3′) | | |
| --- | --- | --- |
| Caspase-3 | Forward | TGGTGATGAAGGGGTCATTTATG |
|  | Reverse | TTCGGCTTTCCAGTCAGACTC |
| BAX | Forward | TGAAGACAGGGGCCTTTTTG |
|  | Reverse | AATTCGCCGGAGACACTCG |
| Bcl2 | Forward | ATGCCTTTGTGGAACTATATGGC |
|  | Reverse | GGTATGCACCCAGAGTGATGC |
| Nrf2 | Forward | AAAGCACAGCCAGCACATTC |
|  | Reverse | GGGATTCACGCATAGGAGCA |
| HO-1 | Forward | AGGTACACATCCAAGCCGAGAAT |
|  | Reverse | CTTGTTGCGCTCTATCTCCTCTT |
| β-Actin | Forward | CCACCATGTACCCAGGCATT |
|  | Reverse | CAGCTCAGTAACAGTCCGCC |
